# Provider Attitudes toward Discussing Fertility Intentions with HIV-Infected Women and Serodiscordant Couples in the USA

**DOI:** 10.4172/2155-6113.1000307

**Published:** 2014-05-13

**Authors:** Lisa Rahangdale, Amy Richardson, Jessica Carda-Auten, Rachel Adams, Catherine Grodensky

**Affiliations:** 1 Department of Obstetrics & Gynecology, University of North Carolina, USA; 2 Center for AIDS Research, University of North Carolina, USA

**Keywords:** HIV, Provider, Fertility, Serodiscordant couples, Women

## Abstract

**Background:**

Recent research suggests that pregnancy is a potentially safe option for couples with at least one HIV-infected adult. Data regarding provider discussion of fertility intentions with women living with HIV (WLWH) or in serodiscordant relationships is limited.

**Methods:**

We conducted a cross-sectional self-administered survey of health professionals who provide HIV services to women in order to assess knowledge and behaviors regarding family planning options for HIV-infected women and serodiscordant couples.

**Results:**

Of 77 respondents, 47(61%) met the inclusion criteria (health care provider who cares for WLWH). Approximately half (57%) of the participants indicated that they always or usually discuss contraception or fertility intentions with their HIV+ female patients of reproductive age. When asked to indicate their awareness of techniques to decrease HIV transmission risk among serodiscordant couples attempting pregnancy, most participants reported awareness of multiple options. Discussion of contraception or fertility intentions was not associated with provider gender, age, and experience in caring for HIV-infected patients, previous training in women’s health or provider’s awareness of options to decrease transmission risk.

**Conclusions:**

HIV providers in this study were knowledgeable of practices that can lead to safer conception and prevent HIV transmission among individuals in serodiscordant relationships but did not always discuss this information with their patients. Further research is needed to explore optimal methods for encouraging such conversations.

## Introduction

As HIV has increasingly become a chronic medical illness and advances in medical care have substantially diminished the risk of mother-to-child HIV transmission (MTCT), [1] more women with HIV desire the cultural and social milestone of motherhood [2–8]. As likelihood of MTCT can now be reduced to less than 2% with antiretroviral (ARV) use during pregnancy, the vast majority of infants born to HIV-infected mothers in the US are not infected with the HIV virus [9,10]. Among a sample of women in HIV care, 65% reported wanting to bear a biological child, yet only 25% reported discussing this with their HIV care provider [11].

For years, HIV care providers have had very little to offer as evidence-based HIV transmission prevention options other than condom use for serodiscordant couples. Since attempting pregnancy requires a couple to discontinue condom use, a desire for pregnancy is in direct conflict with secondary HIV prevention counseling messages. However, recent research indicates that antiretroviral (ARV) use by either an HIV-infected or HIV-negative partner can reduce HIV transmission in serodiscordant heterosexual couples by 96% and 62–73%, respectively [12–14]. With improved options for safe conception and decreased likelihood of MTCT, there is now greater potential for providers and patients to engage in patient-centered conversations on how women living with HIV (WLWH) can both achieve pregnancy safely and avoid perinatal transmission of HIV if desired [15,16]. Organizations such as the Centers for Disease Control and Prevention (CDC) and Infectious Diseases Society of America (IDSA) endorse discussion of fertility intentions and contraception with HIV-infected women [17]. However, it is unclear whether and how issues related to fertility desires and options for safe conception are discussed in the healthcare setting [18–20].

In this study we sought to explore HIV care providers’ knowledge and behaviors regarding family planning options for HIV-infected women and serodiscordant couples.

## Materials and Methods

This was a cross-sectional survey of providers attending a national HIV conference in the Western United States in 2012. This conference was a continuing medical education clinical conference designed for front-line health care professionals providing HIV care for various population groups. The focus was on clinical care and not a presentation of original research. Registered attendees included health care professionals, social workers, researchers and representatives from the health care industry. The survey was distributed in the room where attendees presented for the initial conference session. Inclusion criteria for the analysis included self-report as a health care provider (physicians, nurse practitioners, physician assistants) who reported providing care for WLWH. Providers who reported not having female patients were excluded. Because of its observational and anonymous character, this study was exempted from approval by the University of North Carolina Institutional Review Board.

The survey assessed participants’ professional background and characteristics of their clinical practice and patient panel, using questions adapted from the Centers for Disease Control and Prevention’s Medical Monitoring Project [21]. Participants were asked to indicate how often they themselves or the trainees/care providers under their supervision discussed various health topics (Table 3); with their female HIV-infected patients; answer options included “always,” “often,” “sometimes,” “rarely,” and “completed by other staff in my clinic.”

We also asked participants about their attitudes, knowledge, and barriers to discussion of fertility topics with their HIV-infected patients using survey items developed de novo. Participants were also asked to indicate their attitudes about HIV-infected women desiring pregnancy by selecting their level of agreement or disagreement on a 5-point Likert scale (Table 1).

To assess their knowledge and discussion of options for reducing HIV transmission available to serodiscordant couples desiring pregnancy, participants were asked to indicate which, from a list of options, they had 1) heard about and/or 2) discussed or would discuss with patients (Table 2). We also asked participants to indicate which reasons (Table 4) why they sometimes do not bring up with their reproductive aged female patients the subjects of 1) birth control or contraception and 2) pregnancy intentions or planning.

We calculated descriptive frequencies and means of survey responses. To test for significant bivariate associations (odds ratios) between provider characteristics, attitudes, and knowledge and their discussion of 1) potential pregnancy or fertility intentions and 2) contraception, Fisher’s exact test and Cochran-Mantel-Hanzel tests were used; p<0.05 was statistically significant. SAS version 9.3 (Cary, NC) was used to conduct the analyses.

## Results

### Study sample and participant characteristics

There were 354 individuals registered for the conference, of whom 132 were physicians, nurse practitioners or physician assistants. One hundred twenty-five surveys were distributed during the conference by placing them on number of seats available to attendees of the key note morning seminar. An announcement regarding the survey was made at that time. There is no data on how many individuals presented for the morning seminar. Seventy-seven participants returned surveys, of which 47 met inclusion criteria.

The median age of participants was 51 years (range 25–68) and 49% were women. Fifty-eight percent of those surveyed were physicians, 29% were nurse practitioners, and 17% were physician assistants. Forty-five percent of respondents were board certified in internal medicine, 28% reported infectious disease, 11% reported family medicine, 2% reported pediatrics, and 4% reported other. Forty-five percent did not report a board specialty, 25.5% reported 1 certification, 25.5% reported 2 board certifications, and 4% reported 3 board certifications. Participants reported caring for HIV-infected patients for a median of 11 years (range 0.75 to 30 years). They reported that a median of 90% of their HIV-infected patients were on ARVs (range 22% – 100%) and 10% were women (range 1%–50%).

Seventy-seven percent of participants reported that they provided primary care services to women. When asked about specific primary care services, most stated that they “always” or “often” screened their female patients for lipid profiles (100%), diabetes (94%), colorectal cancer in women aged greater than 50 years (84%), sexually transmitted diseases (94%) and tuberculosis (92%). Eighty-one percent reported referring women who were over the age of 40–50 years for mammography and 67% performed pap smears.

### Knowledge, attitudes, and discussion topics

Providers were asked about their knowledge and attitudes related to reproductive health issues such as contraception and pregnancy in HIV+ women. Table 1 describes providers’ attitudes towards various reproductive health issues in HIV-infected women. Most providers were not comfortable in prescribing contraception themselves, and there was an essentially equal distribution of participants who felt condoms could be used as a single method for both contraception and prevention of acquisition or transmission of infection. Most of the providers said that they “think that it is safe for an HIV+ woman’s health to have a baby”.

When asked to indicate their awareness of techniques to decrease HIV transmission risk among serodiscordant couples attempting pregnancy, most participants reported awareness of sperm-washing (84%) and the following strategies for minimizing risk of transmission during sexual intercourse: maximally suppressed HIV viral load using antiretroviral (ARV) therapy (95%), STD screening and treatment (84%), and PrEP (84%). Many were also aware of other options including management of chronic medical conditions (73%), use of assisted reproductive technology for conception (63%), timed intercourse (54%), post-exposure prophlyaxis (51%), home intrauterine insemination or “turkey baster” method (47%) and fertility evaluation (35%).

Table 2 describes what participants reported as actual discussions of options for reducing HIV transmission risk among serodiscordant couples. The majority of participants reported discussion of the interventions listed above. However, more providers cited knowledge of the topics than discussion in most circumstances.

Table 3 describes specific topics of discussion with HIV-infected female patients regarding their healthcare overall. Participants reported that they “always” or “often” discussed adherence to ARV medication (96%), side effects of ARVs (94%), HIV status of sexual partners (87%), sexually transmitted diseases (81%), and substance use and mental health issues (83%). However, only 57% reported “always” or “often” discussing potential pregnancy or fertility intentions, and though 87% reported discussion of condom use, only 57% reported discussion of contraception. When asked for reasons why these topics may not be discussed, a variety of reasons were offered including time, lack of reproductive aged patients, and that these discussions were the responsibility of other staff (Table 4). Reasons that participants listed out that were not included as survey options regarding discussion of contraception/birth control included the following comments, “Nobody on birth control,” “Always discuss condom use,” “Women of child bearing age are followed in the OBGYN clinic.” Reasons that participants wrote out that were not included as options regarding discussion of pregnancy intentions listed in the survey included the following: “Most HIV + female patients in my practice who are of reproductive age have severe persistent comorbid mental health and/or substance use disorders,” “I do discuss condoms/barrier protection for sexually active patients.” Participants also listed the following comments under both categories"I do this but should revisit it more often,” “Competing priorities,” “Depends on context and reason for visit,” “I see so few reproductive aged women that it sometimes slips my mind.” However, no single reason was identified by the majority of respondents.

### Predictors of engaging in fertility discussions

When comparing participants who said they discussed contraception or fertility intention issues “always” or “often” versus those who discussed these issues “sometimes”"rarely,” or said discussion was done by other staff, there were no significant differences in provider gendercomparing women versus men (OR: 1.00,1.43, p-values 1.00, 0.75 for discussion of contraception and fertility intentions respectively), median provider age (OR:1.80, 1.92, p-values 0.52, 0.75), years’ experience in caring for HIV-infected patients (OR: 1.54, 1.79, p-values 0.54, 0.55), or self-reported previous training in women’s health (OR: 1.17, 2.50, p-values 1.00, 0.21). Similarly, there were no differences in the groups by participants’ level of awareness of options for conception among serodiscordant couples (p-value 0.08, 0.09 for contraception and fertility intentions, respectively). A participants’ level of awareness of options for conception among serodiscordant couples was defined as the self-reported number of options that the participant acknowledged in Table 2.

## Discussion

This is an exploratory study of health care providers’ knowledge and practices regarding several different aspects of reproductive health care for WLWH, as there is little existing research on provider perspectives in regards to these issues. 18 This study demonstrates that providers have a high level of knowledge regarding options for WLWH and serodiscordant couples desiring conception though this is information is not always discussed with patients. A variety of barriers were listed by providers though none stood out as a single point for intervention. There is scant previously published data from the providers perspective to put these findings into context [18,22,23].

Most existing research regarding provider practice in reproductive health care for WLWH comes from the patient perspective [11,19,20]. A survey of 181 women attending an HIV clinic in Baltimore demonstrated that only 67% of women had a general discussion about pregnancy; 31% had a personalized discussion, and the majority of all discussions were patient-initiated [20].

There are multiple interrelated circumstances and influences that determine fertility intentions and behaviors such as contraceptive use in women [24]. Factors such as culture/religion, psychosocial issues, health status, use of ARVs, locus of decision-making, societal stigma and provider attitude are all reported as influences in decisions for pregnancy in WLWH [25,26]. Younger, healthier HIV-infected women often desire pregnancy, and racial norms, partner HIV status and parity have been described as factors associated with desire for fertility.2,5 Male partners’ desires for children are reported as major influences on fertility intentions in WLWH [6,7,26]. Consequently, desire for pregnancy may lead a woman in a serodiscordant relationship to put herself or her partner at risk for heterosexual transmission of HIV in order to achieve his or her fertility desires. Perceived and enacted stigma remain barriers to HIV-infected women considering pregnancy [27–29].

HIV-infected individuals have several options to assist them in becoming pregnant while minimizing risk of HIV transmission to the HIV-negative partner. However, there is a paucity of published research on fertility intentions and decision-making in serodiscordant couples in the U.S [30]. Prior research suggests that some providers may have ethical issues with the provision of assisted reproductive technology for HIV-infected individuals experiencing infertility or desiring safe conception due to potential risks of transmitting HIV to offspring [31]. However, providers in our study did not report any significant ethical or safety concerns regarding conception in WLWH.

This study is limited by its observational nature and small sample size. The lack of statistical association simply may be a reflection of lack of statistical power to find association. Participation was based on a convenience sample; therefore, generalizability may be limited. Most of the participants reported that a minority of their patient population was women, and therefore may not reflect providers with large volumes of WLWH as patients. Valverde et al. reported that of 28% of 666 health care providers surveyed for the US Medical Monitoring Project Provider Survey engaged in preconception counseling with WLWH and that providers who took care of greater than 75% women in their practice were most likely to provide this counseling [18]. However, since women represent only 25% of those infected with HIV in the U.S., [32] most HIV providers that women will encounter will likely care for a minority of women. Additionally, the majority of providers in this survey reported completing other women’s health related prevention measures such as referral for mammograms or pap smear screening so caring for a small number of female patients did not limit these essential primary care decisions in women. Lastly, all of these providers had a specific interest in HIV infection in order to be attending this clinical care conference. One would assume that there level of knowledge may be higher than other HIV care providers who may not seek continuing medical education in HIV-related care.

## Conclusions

HIV-infected men and women and their partners need information about safe methods of achieving pregnancy that minimize risk of transmission to HIV negative partners and HIV-exposed children. Traditional focus on condoms for family planning and prevention of secondary HIV transmission and STIs will not meet the needs of the estimated 280,000 WLWH and 140,000 heterosexual serodiscordant couples living in the U.S.,[16,33]. Both pregnancy and HIV are “sexually transmitted”, (i.e., a consequence of unprotected sexual intercourse), thus secondary HIV prevention counseling must address the inextricably linked issues of fertility and safe conception together [34]. Indeed, simply preventing unintended pregnancy is more cost-effective and more effective at prevention of MTCT than the use of ARV regimens [35].

Though one may argue that specialized centers for care of for WLWH would improve discussion of reproductive needs, this practice would not be feasible for women living long distances from specialty centers and would not address the needs of HIV-infected men who desire children. Furthermore, this study did not demonstrate an association with knowledge, training or provision of other female-focused preventative health services with actual provision of these services. Additionally, providers in this survey reported providing other female-focused primary care services such as pap smear screening and referral for mammography despite women comprising a minority portion of their practice. As reproductive health counseling is an essential part of primary care for women and an interest in childrearing increases as HIV has become a chronic illness, further research is needed to explore optimal methods for encouraging conversations and providing training for such conversations between providers and patients regarding options for safe pregnancy in people living with HIV in the United States.

## Figures and Tables

**Table 1:** Provider attitudes towards topics related to contraception, pregnancy and fertility issues in HIV+ women, n=46.

Statement	Strongly agree (or) Agree n(%)	Neither agree (or) disagree n (%)	Disagree (or) Strongly disagree n (%)
Condoms alone are sufficient to prevent STD acquisition, HIV transmission and pregnancy.*	20(43)	4(9)	21(45)
I feel comfortable prescribing hormonal contraception.	14(30)	14(30)	18(39)
I think it is safe for an HIV+ woman’s health to have a baby.	41(88)	5(11)	0(0)
I think it is morally wrong for an HIV + woman to have a baby.	0(0)	2(4)	44(93)

* Missing response=1

**Table 2:** Reported discussion of options for serodiscordant heterosexual couples interested in conception (more than one option could have been chosen), n=47.

Option	HIV+ female HIV-negative male n (%)	HIV+ male HIV-negative female n (%)
I discourage these couples from pregnancy	0	0
There are no options for these couples	0	0
Maximally suppressed viral load using ARV therapy	42(89)	41(87)
Management of any chronic medical conditions	33(70)	32(68)
Screening and treatment for STDs	36(77)	35(75)
Pre-exposure prophylaxis (PreP)	34(72)	31(66)
Post-exposure prophylaxis	22(47)	20(43)
Timed intercourse	20(43)	19(40)
”Turkey baster” (home intrauterine insemination)	14(30)	NA
Sperm Washing	NA	30(64)
Fertility evaluation	13(28)	11(23)
Referral to specialist for assisted reproductive technology	27(57)	24(51)
Office intrauterine insemination	1(2)	0
Adoption	1(2)	1(2)
Referral to high risk OB/GYN	1(2)	0
None of the above	2(4)	0

NA=not applicable

**Table 3:** Frequency of discussion of various HIV-related and women’s health topics by health provider or other staff with HIV-infected female patients, n=47.

Health Topic	Always (or) Often n (%)	Sometimes (or) Rarely n (%)	Missing Response n (%)
Adeherence to antiretrovirals	45 (96)	1(2)	1(2)
Side effects of antiretrovirals	44(94)	2(4)	1(2)
Potential pregnancy or fertility intentions	27(57)	17(36)	3(6)
Contraception	27(57)	17(36)	3(6)
Condom use	40(87)	4(9)	3(6)
HIV status of sexual partners	41(87)	4(9)	2(4)
Sexually transmitted diseases prevention	38(81)	6(13)	3(6)
Substance abuse	39(83)	7(15)	1(2)
Mental health issues	40(85)	6(13)	1(2)

**Table 4:** Reasons acknowledged by providers for not discussing contraception or pregnancy intentions, n=47.

Reason for not discussing	Birth Control/ Contraception n (%)	Pregnancy intentions (or) planning n (%)
I always bring up contraception/pregnancy with my reproductive aged HIV+ female	16 (34)	14 (30)
Not enough time	9(19)	9 (19)
Do not feel comfortable with	1 (2)	4 (9)
Do not have adequate training on the topic	4(9)	3 (6)
Not my job	0(0)	1 (2)
Other members of my health care team are assigned to discuss the issue	6 (13)	6 (13)
My patients are not interested in this topic	2 (4)	3 (6)
None of my female HIV+ patients are reproductive aged	8 (17)	9 (19)
None of my female patients are sexually active with men	2 (4)	2 (4)
It is against my belief/morals to discuss birth control/pregnancy	1 (2)	0 (0)
Never occurred to me	1 (2)	2 (4)
Condoms provide adequate birth control	0 (0)	NA
Pregnancy is too risky for the health of HIV+ women	NA	0 (0)
The risk of transmission to her partner is too high	NA	0 (0)
The risk of transmission to her baby is too high	NA	0(0)
Other	9 (19)	7 (15)

NA= not applicable
